# Prenatal Breastfeeding Education with or without Hand Expressing Human Milk and Breastfeeding Duration in a Rural Population

**DOI:** 10.3390/nu16193303

**Published:** 2024-09-29

**Authors:** Farjana Zaman, Shannon Morgan, Cheryl Scalora, Marcia Nelson, Jimi Francis

**Affiliations:** 1 College for Health, Community, and Policy, University of Texas at San Antonio, San Antonio, TX 78249, USA; farjana.zaman2021@gmail.com; 2 Nacogdoches Women’s Center, Nacogdoches, TX 75965, USA

**Keywords:** colostrum, prenatal breastfeeding education, breastfeeding outcomes, hand expressing human milk

## Abstract

Background: The benefits of breastfeeding are well recognized. However, exclusive breastfeeding (EBF) rates are well below the Healthy People 2030 Guidelines, with a rapid drop in exclusive breastfeeding over the first 3 months of life. Although breastfeeding support has increased the initiation of breastfeeding, the role of perinatal interventions, particularly in the context of breastfeeding support, remains a topic of contradiction. Methods: This observational study was designed to assess the impact of a unique prenatal educational intervention in rural East Texas. The study had two aims: (1) to determine whether the addition of prenatal breastfeeding education increased the rate of exclusive breastfeeding at four time points, and (2) to assess the impact of hand expression education on breastfeeding exclusivity. Results: Differences in breastfeeding behaviors were seen between those who received the education and those who did not for exclusivity and duration of breastfeeding. Participants who received the hand expression education were 1.79 times more likely to continue EBF practices at three months postpartum, 6.27 times more likely to continue EBF practices at six months postpartum, and 3.34 times more likely to continue breastfeeding at twelve months postpartum than those who did not receive any hand expression education. Conclusion: This study demonstrates that prenatal education is beneficial for increasing breastfeeding exclusivity and duration and underscores its potential to improve maternal and infant health outcomes. Further research is needed to resolve the ongoing debate and advance our understanding of interventions to increase breastfeeding duration.

## 1. Introduction

It is well known and well documented that breastfeeding is essential to providing human milk to infants. Human milk is an excellent product that provides optimal nutrition, which allows for the healthy growth and development of infants [1]. Exclusive breastfeeding in the first six months of life serves as the infant’s ideal source of nutrition by providing essential vitamins [2], minerals [3], and protein [4]. Breastfeeding provides short-term and long-term benefits to both mother and baby [5,6]. Getting a good start at breastfeeding with the consumption of colostrum is crucial. The role and importance of colostrum feeding in newborns for their growth and development are well documented. Colostrum is a rich source of protein, carbohydrates, and vitamins. This early feeding practice is beneficial and crucial for the infant’s health and development [7]. Colostrum has a natural laxative effect, which removes soft stool (meconium) from the system, reducing the risk of jaundice [8]. Colostrum has higher concentrations of immunoglobulins, lactoferrin, and lysozyme than mature milk, which provides crucial immune protection for infants at risk of infections [9].

With its unique [10] and immune-boosting benefits [11], colostrum is crucial to a baby’s early nutrition. Breastfeeding during the first few days of life is essential to ensure the infant receives these critical benefits. However, in the first hours after an infant’s birth, they are often fed infant formula for various reasons [12], including hypertension interventions and C-section birth [13]. Initiating breastfeeding within the first hour of life, frequently called the “Golden Hour [14]”, is crucial for both mother and baby [15]. Research shows that neonates who breastfeed within the first hour are more likely to continue exclusively breastfeeding for the recommended six months, significantly reducing the risk of infant mortality. This underscores the urgent need to promote breastfeeding for the health and well-being of infants. Unfortunately, exclusive breastfeeding has continued to decline [16].

Prenatal hand expressing of colostrum has gained attention as a proactive practice for mothers, particularly those with conditions like gestational diabetes or those expecting preterm or cesarean births. Current research suggests that hand expressing colostrum from around 36 weeks of pregnancy is generally safe for most women with uncomplicated pregnancies [17]. Studies indicate that prenatal hand expression does not significantly increase the risk of preterm labor or other adverse pregnancy outcomes as long as it is practiced under the guidance of a healthcare provider [18]. Furthermore, collecting colostrum prenatally can provide a valuable nutritional resource for newborns [19], especially those facing feeding challenges immediately after birth. But can prenatal hand expressing of colostrum increase the duration and exclusivity of breastfeeding?

## 2. Materials and Methods

This study evaluated the impact of adding a novel breastfeeding education module to perinatal care on breastfeeding outcomes. Two education modules were offered: one with generalized breastfeeding education and one teaching hand expressing of colostrum.

### 2.1. Setting and Participants

This study was conducted in rural East Texas, in the United States of America, a region with a significant need for improved breastfeeding outcomes. The rate of exclusive breastfeeding (EBF) in this region at six months of age is 23.9%, far below the Healthy People objective of 42.4% [20]. The study participants were women attending the local clinical women’s health center for their perinatal care.

### 2.2. Education

All expectant mothers receiving prenatal care at a midwifery practice were asked about their infant feeding preference(s) and plans for feeding at 28–30 weeks’ gestation. All mothers interested in breastfeeding were invited to attend an in-office 45 min lactation education class before 34 weeks’ gestation. Breastfeeding education was not mandatory for the patients at this clinic. Module one included primary breastfeeding education. The participants in module two were taught how to hand express, collect, and store their colostrum at 37–38 weeks’ gestation. Patients who collected their colostrum were instructed to take it to the hospital in frozen 1 mL syringes at the time of labor or scheduled delivery day. The colostrum, which would be used if the baby could not be fed directly at the breast immediately after birth, was provided to hospital staff at hospital admission. The hospital required the human milk to be labeled with the patient identification number and stored following the hospital’s human milk handling policy. Data from the patient charts were collected by the clinic’s staff and placed in a spreadsheet. These data were de-identified by the clinic’s staff before being given to the researchers.

### 2.3. Survey

Additional data were collected via a survey questionnaire distributed to patients by the clinic’s staff. Data from the surveys were matched with the chart data by the clinic’s staff to preserve de-identification. The patients with chart data and a completed survey were included in the final data set. Clinical staff with legal access to patient data gave each patient a unique identification number and de-identified the data. The de-identified and coded data were recorded on a spreadsheet before the research team conducted data analysis.

The questionnaire contained information on EBF duration (1 month, 3 months, 6 months, and 12 months postpartum), any feeding issues, breastfeeding within the first hour of birth, feeding mode, whether the baby was placed in the NICU, the type of breastfeeding education they received, whether the mother received lactation support, whether the mother received education on hand expression of colostrum, whether colostrum was taken to hospital, and whether colostrum was fed to the infant in the hospital before discharge.

The University of Texas at San Antonio’s Institutional Review Board evaluated the project. Due to the use of de-identified data, it was classified as “not regulated research”. The survey portion of the data was voluntary, so consent was implied if the participant completed it.

### 2.4. Statistics

The variables of interest were evaluated using descriptive statistics. For categorical data analysis, crosstabulation analysis was conducted for hand expressing education, prenatally expressing colostrum, the infant being fed colostrum before hospital discharge, the infant being breastfed in the first hour of life, infant weight gain issues within the first 12 months of life, mothers who received lactation education, and mothers who received lactation support.

## 3. Results

In the study, 178 de-identified patients returned their questionnaires, and the corresponding data were included for analysis. The demographics of the respondents are shown in Table 1. Of the 178 respondents, 89% intended to breastfeed, and 50.6% (*n* = 90) did not receive prenatal or hand expression breastfeeding education. The remaining 49.4% (*n* = 88) received in-person prenatal hand expression of milk education (e.g., prenatal colostrum expression and/or colostrum feeding in hospital) as part of the novel breastfeeding education intervention program. All participants in the hand expression group could express, but not all took colostrum to the hospital at the time of birth. None of those who hand expressed colostrum had preterm labor due to hand expressing.

As shown in Table 2, 50 of the 178 respondents who received hand expression education took their colostrum to the hospital, with 38 of the infants receiving it. Of those who did not receive hand expression education, 23 took expressed colostrum and took it to the hospital, with three of the respondents’ babies receiving colostrum in the hospital (Chi-square *p*-value 0.001).

Table 3 shows the data for breastfeeding at each time point with lactation education and hand expression education. Exclusive breastfeeding occurred up to 6 months of age, with complementary foods added after that. Hand expression education was significantly associated with a longer duration of breastfeeding over 12 months. Those who received hand expression education had a higher rate of EBF at three months, six months, and twelve months than those who did not receive hand expressing education categorized by parity.

When categorized by parity, as shown in Table 4, at each time point, exclusive breastfeeding was more common among primiparous women who received education than it was among those who did not. This effect continued as time passed, with the primiparous women who received the hand expressing education continuing to breastfeed for more extended periods. Hand expression education was significantly associated with a longer duration of breastfeeding over 12 months.

Respondents who breastfed their infants in the first hour of birth were more likely to continue EBF for a longer period (e.g., 6 months or 12 months rather than 3 months) than those who did not breastfeed their babies in the first hour of life (*p*-value 0.001). Of those who did not receive hand expressing education, 65.7% of respondents breastfed their infant within the first hour of life, compared to 78% who did receive the education. Breastfeeding during the first hour of birth is more generalized to the respondents. Lastly, the observed Chi-Square statistic (X^2^) is 3.185, with a *p*-value of 0.052 (not significant).

The respondents who received the hand expression education were 1.79 times more likely to continue EBF practices at three months postpartum, 6.27 times more likely to continue EBF practices at six months postpartum, and 3.34 times more likely to continue breastfeeding at twelve months postpartum than those who did not receive any hand expression education.

Overall, 41% of the respondents received no lactation assistance after the birth of their infants. Of the respondents polled, 27% received lactation assistance in the hospital before discharge, while 25.8% received lactation assistance at the clinic, and 6.2% received lactation assistance from a community lactation consultant. Only 32% of respondents received lactation assistance outside of the hospital. Of those who did not receive assistance but did have hand expressing education, 33% were still breastfeeding at 6 months postpartum compared to 25.5% who did not receive the education. At the 12-month time point, regarding the group that did not receive lactation assistance, 16.3% of those breastfeeding had received hand expressing education, compared to 11.8% who did not receive the education (*p*-value = 0.013).

## 4. Discussion

While the breastfeeding initiation rate is high in Texas among the WIC population, with initiation now at 89% [21], which matched the breastfeeding intentions of the respondents in this study, it is known that exclusive breastfeeding declines in the study locale due to a lack of resources in a rural environment. Increased challenges occur with continuing breastfeeding practices at the 3-month, 6-month, and 12-month postpartum periods, particularly in rural areas, where lactation assistance is scarce [22].

The importance of EBF cannot be overstated. Exclusive breastfeeding, i.e., feeding directly from the breast, is vital for providing essential nutrients for infant growth and development [23]. In addition, EBF strengthens the immune system, as human milk contains antibodies that help protect the baby against infections and illnesses [24]. EBF also promotes bonding between mother and baby [25].

Our findings support the premise that breastfeeding education before birth can help increase breastfeeding duration; as we have seen in clinical practice, breastfeeding empowers mothers to become familiar with their breasts, providing them with the necessary knowledge and skills to overcome possible challenges. As shown in this study, prenatal breastfeeding education can increase breastfeeding duration through improving the awareness of breastfeeding benefits. Breastfeeding education can help mothers understand the benefits of breastfeeding for both the baby and the mother. When mothers are aware of the positive effects of breastfeeding on their child’s health, growth, and development, they are more likely to commit to breastfeeding for a longer duration. Prenatally, breastfeeding education can increase one’s understanding of milk production and what factors affect milk supply [26]. This knowledge can help them manage engorgement, low milk supply, or overproduction. Through breastfeeding education, mothers can become better prepared for common breastfeeding challenges, such as sore nipples, latch difficulties, and mastitis [27]. When mothers are prepared for these challenges through knowledge, they know where to find support and assistance. Lactation consultants offer specialized guidance to resolve breastfeeding challenges and milk supply issues [28]. Prenatal breastfeeding education can also inform mothers with respect to where to seek help and support if they encounter difficulties. Access to this support can help mothers overcome challenges and continue breastfeeding. Unfortunately, mothers in rural areas often have fewer resources than mothers in urban settings [29,30,31].

This study demonstrates that prenatally expressing colostrum is a powerful tool that can empower mothers with little or no lactation assistance. Prenatally expressing colostrum can help reassure these mothers that they have a good supply of colostrum ready for their baby’s first feeds. In addition, learning to express their milk by hand can boost a new mother’s confidence in providing milk for her baby. By learning how to hand express to collect colostrum, mothers can gain confidence in their ability to produce milk and overcome concerns about breastfeeding. The findings in this study substantiate the benefits of this practice, which previously were unsubstantiated.

Prenatal expression of colostrum can comfort a new mother and potentially increase her feelings of self-efficacy [32], especially important when unusual circumstances arise, such as a medical condition that may require separation from their baby after birth, such as gestational diabetes [33] or COVID-19 [34]. In these situations, having a supply of colostrum on hand can help ensure that the baby receives the essential nutrients and antibodies they need until the baby can be reunited with their mother. In summary, the findings reported in this study are clinically significant and could play a role in improving breastfeeding outcomes and increasing breastfeeding duration.

## 5. Conclusions

Our study has found a consistent positive relationship between breastfeeding practices and prenatal education. We also conclude that hand expression education is helpful in rural areas, where resources for antenatal care may be limited. This study’s results could significantly impact the health of mothers and infants in this region, highlighting their importance and significance. Additional research is needed to advance our understanding of breastfeeding challenges, including how to prevent and/or treat them in varied areas and populations.

## Figures and Tables

**Table 1:** Demographics by lactation education and hand expression education.

		Received Lactation Education			Received Hand Expression Education		
		No	Yes	Total	No	Yes	Total
Maternal Age	Mean Age in Years	24	25		24	25	
Marital Status	Unmarried	30	29	59	38	21	59
	Married	60	59	119	67	52	119
Education	Less than High School	36	13	49	41	8	49
	High School Graduate	34	26	60	39	21	60
	Some college	5	20	25	7	18	25
	College Graduate	14	27	41	17	24	41
	Postgraduate	1	2	3	1	2	3
Race	Hispanic	16	14	30	21	9	30
	Black	19	23	42	21	21	42
	White	52	51	103	60	43	103
	Other	3	0	3	3	0	3
Income	<$30,000	57	47	104	67	37	104
	>$30,000 to $74,999	30	34	64	34	30	64
	>$75,000	3	7	10	4	6	10
Parity	Primiparous	50	85	135	64	71	135
	Multiparuos	40	3	43	41	2	43

**Table 2:** Crosstab distribution of hand expression education vs. colostrum taken to hospital.

	Hand Expression Education			
Colostrum taken to hospital	*N* = 178	**No**	**Yes**	**Total**
	No	90 (50.6%)	15 (8.4%)	105 (59%)
	Yes	23 (13%) [3 Fed Colostrum]	50 (28%) [38 fed colostrum]	73 (41%)
	Total	113 (63.5%)	65 (36.5%)	178 (100%)
(*p*-value = 0.001)				

**Table 3:** Crosstab distribution of hand expression education’s impact on breastfeeding practices in the first 12 months of life.

Hand Expressing Education		1 Month		3 Months		6 Months		12 Months	
		No	Yes	No	Yes	No	Yes	No	Yes
	No	2	103	13	92	58	47	76	29
	Yes	0	73	0	73	12	61	32	41
Total		2	176	13	165	70	108	108	70
Lactation Education	No	2	88	12	78	54	36	69	21
	Yes	0	88	1	87	16	72	39	49
Total		2	176	13	165	70	108	108	70

**Table 4:** Crosstab distribution of hand expression education’s impact on breastfeeding practices in the first 12 months of life categorized by parity.

Hand Expressing Education			Bf1month		Total	BF3month		Total	BF6month		Total	BF12month		Total
			No	Yes		No	Yes		No	Yes		No	Yes	
No	Parity	Primiparous	2	62	64	12	52	64	45	19	64	51	13	64
		Multiparous	0	41	41	1	40	41	13	28	41	25	16	41
	Total		2	103	105	13	92	105	58	47	105	76	29	105
Yes	Parity	Primiparous	0	71	71	0	71	71	11	60	71	30	41	71
		Multiparous	0	2	2	0	2	2	1	1	2	2	0	2
	Total		0	73	73	0	73	73	12	61	73	32	41	73
Total	Parity	Primiparous	2	133	135	12	123	135	56	79	135	81	54	135
		Multiparous	0	43	43	1	42	43	14	29	43	27	16	43
	Total		2	176	178	13	165	178	70	108	178	108	70	178

## Data Availability

The original contributions presented in the study are included in the article, further inquiries can be directed to the corresponding author/s.
